# Investigating the relationship between spousal violence against women and total fertility rate in Afghanistan

**DOI:** 10.1186/s12889-024-18944-6

**Published:** 2024-05-31

**Authors:** Mehri Shams Ghahfarokhi

**Affiliations:** Department of Social Sciences, University of Isfahan, Isfahan, Iran

**Keywords:** Fertility, Spousal violence against women, Intimate partner violence, Parity progression ratios, Mean closed birth intervals

## Abstract

**Background:**

spousal violence against women (SVAW) is a common form of violence that occurs within the family context, with spouses being the main perpetrators. Afghanistan has one of the highest rates of SVAW in the world, and its impact on reproductive health and fertility is not well understood. This study aims to investigate the extent to which SVAW influences the total fertility rate (TFR) of Afghan women.

**Methods:**

In this study, a regression model of discrete-time survival models was used to calculate the total fertility rate (TFR), parity progression ratio (PPRs), and average closed birth intervals (CBI) between two children. The method used in this study has its roots in the works of Griffin Finney (1983) and was further developed by Redford et al. (2010). The study population utilized the 2015 Afghanistan Demographic and Health Survey, and sample weights were used to ensure accurate estimates for the population of Afghanistan as a whole.

**Results:**

The study found that women in Afghanistan who have experienced SV are more likely to progress to the next parity, start childbearing faster, and continue to do so. Women who have not experienced SV tend to progress to higher parities at a slower pace during their initial reproductive years. The study also suggests that women with spousal violence (SV) experience may have slightly higher fertility rates and shorter birth intervals for certain birth orders, although the differences between the two groups are generally small. Specifically, the total fertility rate (TFR) for women who experienced SV was 6.9, while the TFR for women who did not experience SV was 6.2.

**Conclusions:**

These results provide valuable information for policymakers and public health professionals in developing effective policies and programs to address SVAW and improve maternal and child health outcomes in Afghanistan.

**Supplementary Information:**

The online version contains supplementary material available at 10.1186/s12889-024-18944-6.

## Background

Spousal violence against women (SVAW) is a prevalent and significant form of violence that takes place within the context of marriage or intimate relationships, with spouses being the primary perpetrators. This kind of violence can take a variety of forms, such as physical, psychological, and sexual abuse, whether it occurs in public or private places. Spousal violence against women is also referred to as marital violence or spousal abuse.

Spousal violence against women and domestic violence have distinct conceptual differences. domestic violence encompasses various forms of abuse, such as elder abuse, sibling abuse, child abuse, spousal abuse, and parent abuse. It can involve individuals who have an intimate relationship but may not live together. In contrast, SVAW is a specific form of domestic violence that specifically pertains to aggression between spouses or intimate partners [1].

Globally, at least 27% of women aged 15–49 who are married or in a relationship have experienced physical and/or sexual violence committed by an intimate partner at least once in their life. However, Afghanistan stands out among the 19 countries with the highest rates of such violence, with 46% of women affected [2]. Despite the increase in frequency and severity of this issue, it has not received much attention or concern, due to cultural reasons unique to Islamic countries [3]. Despite the passage of the law on the elimination of violence against women by the Afghan government in 2009 [4], its impact has been devastating due to the lack of a strong central government, ongoing insecurity, and insufficient enforcement of laws and regulations. These factors have contributed to the marginalization of women in Afghanistan.

SVAW has been shown to have significant health consequences for women, including exacerbating menopausal symptoms, heightening the likelihood of developing diabetes and sexually transmitted infections, and leading to high-risk behaviors such as substance and alcohol abuse, as well as developing chronic illnesses and pain [5]. Furthermore, spousal violence is a cause for concern regarding reproductive rights, which are assessed globally using reproductive health indicators as the primary measure of success in achieving these rights [6]. These indicators include the entitlement to regulate fertility, which includes the power to decide whether or not to have children, as well as the authority to determine the timing and number of children [7].

The rapid population growth rate in Afghanistan has created major challenges in achieving the targets of the sustainable development goals (SDGs) [8]. Progress towards SDG 3.7 (sexual and reproductive health of women) and SDG 5 (gender equality) can contribute to slowing Afghanistan’s population growth [9]. It is currently experiencing a population growth rate of around 2.6% and a total fertility rate of 4.3 children per woman [10], which places it among the countries undergoing a baby boom. To control population growth, it is necessary to reduce fertility, and to achieve this, it is crucial to identify the factors that contribute to either an increase or a decrease in fertility rates and to develop strategies to address them.

The effects of SVAW on fertility are not always well-defined or consistent, particularly in developing countries, where both SVAW and TFR are high, despite some evidence supporting a link between violence and fertility [11–13]. Research has revealed that in certain circumstances, there is a connection between violence and fertility. One such study is the world health organization’s multi-country investigation into women’s health and violence, which took place in 15 sites across 10 countries [14]. This study found that, except for Thailand city and Japan city, women who had encountered violence were inclined to have more children than women who had not experienced abuse. However, other studies have not found any association between violence and fertility or have suggested that they are only linked to particular circumstances [15]. These findings suggest that the relationship between violence and fertility may be complex and context-specific, and further research is necessary to gain a complete understanding of this association and its effects on reproductive health. Moreover, most studies have only measured the relationship between these variables, without specifying the degree of difference between them.

This research aim is to investigate the indicators of reproductive rights within Afghanistan’s healthcare system. Reproductive coercion and abuse violate reproductive rights, as they involve the use of power and control to interfere with an individual’s ability to make decisions about their reproductive health. Reproductive coercion and abuse are particularly prevalent in situations of intimate partner violence, where abusers may use reproductive control as a tool to maintain power and control over their partners. The world health organization recognizes reproductive coercion as a form of intimate partner violence and recommends that it be addressed as part of comprehensive efforts to prevent and respond to violence against women [16].

Fertility, birth spacing, and parity progression ratios are important indicators for understanding women’s and children’s reproductive health. High fertility rates and parity progression ratios increase the risk of poor maternal and child health outcomes, such as maternal mortality, infant mortality, and low birth weight [17–19]. Short birth intervals can also lead to negative perinatal outcomes and maternal depletion syndrome [19–21]. By examining these indicators, policies and interventions can be developed to improve maternal and child health outcomes.

Afghanistan was chosen for several reasons, including its high TFR of 5.3 children per woman and low rate of contraceptive use at 23% [22]. These factors make it easier to detect the covariation between SVAW and TFR than in industrial societies, where sexual activity and childbearing are often separate. This study is significant as it aims to clarify how spousal violence is related to fertility, enabling policymakers to make informed decisions about population growth rates and implement effective birth control policies and programs. Apart from shedding light on how intimate partner violence affects reproductive behavior, this investigation may enhance women’s status within both society and the family, emphasize their vital role, and expedite various initiatives, including demographic policies. Therefore, this research aims to answer the following inquiry: To what extent is the TFR of Afghan women influenced by spousal violence (SV)? Does the experience of SV affect the likelihood of having higher birth orders? And does the experience of SV change the average closed birth intervals (CBI)?

## Methods

The method used to calculate the total fertility rate (TFR), parity progression ratio (PPRs), and average time interval between two children was a regression model of discrete-time survival models for each parity, using the complementary log-log model. The method used in this study has its roots in the works of Griffin Finney (1983). By using the probabilities of marriage and fertility in a given period, Finney created life tables for each of the transition periods mentioned, producing indicators such as the PPR, the average age of marriage, and the mean CBI [23].

However, while the Finney method is accurate in measuring fertility, it does not allow for multivariate analysis of total fertility. The next step in the development of this method was taken by Redford et al. (2010), who used survival regression models to estimate the marriage and fertility probabilities required to construct life tables. It should be noted that the results of evaluating the multivariate method of estimating fertility indices based on data from India and the Philippines indicate its good validity [24–26]. All statistical analyses were carried out through the utilization of Stata 13 software [27].

Parity refers to the increase in the number of children and the time interval between two consecutive events related to childbearing in a row in the regression model formula. For example, the interval between marriage (first event) and the birth of the first child (second event) is called the transition from marriage to the first child (M-1), and the interval between the birth of the first child (first event) and the birth of the second child (second event) is called the transition from the first to the second birth (1–2). The last parity includes the period from the birth of the last child to 10 years after the birth of the last child. The last transition period includes the period from the birth of the last child to usually 10 years after the birth of the last child, as the probability of having the next child 10 years after the birth of the last child is very low. If a person is under the age of 50 at the end of this period, the next stage of the analysis is skipped due to the almost zero probability of fertility. If a woman reaches the age of 50 before the end of the 10-year transition period, this period will be shorter for her.

In summary, each woman’s life cycle from the age of 15 to 49 is divided into different periods of transition, and each period is analyzed separately. Additionally, if a person does not get married by the age of 40, she is excluded from the analysis due to the low probability of marriage after that age. The regression models used are a set of probabilities of marriage or childbirth by parity (i), and duration in parity (t). Using these probabilities, multidimensional marriage and fertility life tables are created, and fertility and marriage indexes are extracted from these tables.

To fit regression models for the birth-to-first-marriage (B-M) transition, data needs to be transformed into person-year data. This involves expanding the number of cases for each woman in the data to the number of year units she is present in each extended transition period. The variable DUR measures the time interval between the first and second events and represents the number of times a woman is present in the data for each transition period. The variable t represents the time interval of each case for each person from the beginning of the transition period. After preparing the data, the probability of having the first child is calculated for both uncontrolled and controlled groups using a specific formula (The same formula is applied to the remaining transitions, with the only difference being that the data is prepared separately for each transition).

The probability of having the first child in the uncontrolled group is defined as follows:

$$\eqalign{{{\rm{P}}_1} = & {\rm{ }}1 - {\rm{exp }}\{ - {\rm{exp}}\,[{{\rm{b}}_0} + {\rm{ (}}\left( {{{\rm{b}}_1}\,{\rm{year}}} \right) \cr {\rm{ }} & + {\rm{ }}\left( {{{\rm{b}}_2}\,{\rm{yea}}{{\rm{r}}^2}} \right){\rm{ }} + {\rm{ }}\left( {{{\rm{b}}_3}\,{{\rm{X}}_1}} \right) + {\rm{ }}({{\rm{b}}_4}\,{{\rm{X}}_1} \times {\rm{year}}) \cr {\rm{ }} & + {\rm{ }}({{\rm{b}}_5}\,{{\rm{X}}_1} \times {\rm{yea}}{{\rm{r}}^2})) \cr}$$

And the probability of having the first child in the controlled group is defined as follows:

$$\eqalign{{{\rm{P}}_{\rm{1}}}{\rm{ = }} & {\rm{ 1 - exp \{ - exp[}}{{\rm{b}}_{\rm{0}}}{\rm{ + ((}}{{\rm{b}}_{\rm{1}}}\,{\rm{year) + (}}{{\rm{b}}_{\rm{2}}}\,{\rm{yea}}{{\rm{r}}^{\rm{2}}}{\rm{)}} \cr & {\rm{ + }}\left( {{{\rm{b}}_{\rm{3}}}\,{{\rm{X}}_{\rm{1}}}} \right){\rm{ + (}}{{\rm{b}}_{\rm{4}}}\,{{\rm{X}}_{\rm{1}}}{\rm{ \times year) + (}}{{\rm{b}}_{\rm{5}}}\,{{\rm{X}}_{\rm{1}}}{\rm{ \times yea}}{{\rm{r}}^{\rm{2}}}{\rm{)}} \cr & {\rm{ + }}\left( {{{\rm{b}}_{\rm{6}}}\,{{\rm{X}}_{\rm{2}}}{\rm{ + }}{{\rm{b}}_{\rm{7}}}\,{{\rm{X}}_{\rm{3}}}{\rm{ + }}{{\rm{b}}_{\rm{8}}}\,{{\rm{X}}_{\rm{4}}}{\rm{ + }}{{\rm{b}}_{\rm{9}}}\,{{\rm{X}}_{\rm{5}}}} \right){\rm{)}} \cr}$$

Where P_1_ is probability of fertility; b_0_ is intercept; X_1_ is independent variable; X_2_…X_5_ are control variables; b_1_…b_9_ are coefficients of independent and control variables; year is year;

year^2^ is year to the power of 2; year × X_1_ is interaction of year with independent variable; year^2^ × X_1_ is interaction of year to the power of 2 with independent variable.

Fertility life tables can be created based on the estimated values of P_it_. Both the life tables and the indexes calculated from these tables are multivariate. Multivariate means that life tables can be calculated separately according to different classifications of a variable. The life tables have four quantities: ${\text{P}}_{\text{t}}$, which represents the conditional probability of risks (e.g., first or subsequent child) between time t and t + 1; ${\text{S}}_{\text{t}}$, which represents the number of survivors at time t (who have not yet experienced subsequent child); ${F}_{\text{t}}$, which represents the number of births between time 0 and t; and ${F}_{\text{t}}$, which represents the number of risks between time t and t + 1.

The mathematical formulas for B-M transitions in life tables are as follows:

1 $${{\rm{S}}_0} = 1000$$

2 $${{\rm{S}}_{\rm{t}}} = {{\rm{S}}_{{\rm{t - 1}}}}\left( {1 - {{\rm{P}}_{t{\rm{ - 1}}}}} \right)\,\,\,\,\,\,\,{\rm{for}}\,{\rm{t}}\,{\rm{ > }}\,{\rm{0}}$$

3 $${{\rm{F}}_{\rm{t}}}{\rm{ = 1000}}\,{\rm{ - }}\,{{\rm{S}}_{\rm{t}}}$$

4 $${{\rm{f}}_{\rm{t}}}{\rm{\, = \,\,}}{{\rm{S}}_{\rm{t}}}\,\,{{\rm{P}}_{\rm{t}}}$$

The non-conditional probability of risk at time t is obtained by dividing ${F}_{\text{t}}$ by 1000. The non-conditional probability of risk at the end of the life table is the proportion of progress to the first child (PPR) for the transition from marriage to first child. For M-1 transition, the time interval in the life table is 10 years (from 0 to 9) and the formula for PPR is:

5 $${\rm{PPR = }}\,{{\rm{F}}_{{\rm{10}}}}{\rm{/1000}}$$

6 $${\rm{Mean}}{\mkern 1mu} {\rm{\,closed}}{\mkern 1mu} {\rm{\,interval = }}\sum {[({{\rm{f}}_{\rm{t}}}{\rm{/}}{{\rm{F}}_{{\rm{10}}}}{\rm{)(t)}}]}$$

Where P_1_ is probability of fertility; b_0_ is intercept; X_1_ is independent variable; X_2_…X_12_ are control variables; b_1_…b_16_ are coefficients of independent and control variables; year is year;

year^2^ is Year to the power of 2; year × X_1_ is interaction of year with independent variable; year^2^ × X_1_ is interaction of year to the power of 2 with independent variable.

To fit regression models for the transition period from the first to the second child, data must be prepared. Only women who have at least one child can enter the data, and women without children are removed from the data. After preparing the data for each parity up to 10 children and beyond, it can be used to fit the models. Life tables are created for each transition period to calculate the increase in the number of children in each period. The PPR is calculated for each transition period, including B-M, M-1, 1–2, 2–3, 3–4, 4–5, 5–6, 6–7, 7–8, 8–9, 9–10+. The closed interval between births is also calculated for these transition periods. The total fertility rate is calculated using the parity progress ratios [24].

$$\eqalign{{\rm{TF}}{{\rm{R}}_{{\rm{PPR}}}} = & {{\rm{P}}_{\rm{M}}}{\rm{ + }}{{\rm{P}}_{\rm{M}}}{{\rm{P}}_{\rm{1}}}{\rm{ + }}{{\rm{P}}_{\rm{M}}}{{\rm{P}}_{\rm{1}}}{{\rm{P}}_{\rm{2}}} & \cr & + {{\rm{P}}_{\rm{M}}}{{\rm{P}}_{\rm{1}}}{{\rm{P}}_{\rm{2}}}{{\rm{P}}_3} + {{\rm{P}}_{\rm{M}}}{{\rm{P}}_{\rm{1}}}{{\rm{P}}_{\rm{2}}}{{\rm{P}}_3}{{\rm{P}}_4} \cr & + {{\rm{P}}_{\rm{M}}}{{\rm{P}}_{\rm{1}}}{{\rm{P}}_{\rm{2}}}{{\rm{P}}_3}{{\rm{P}}_4}{{\rm{P}}_5} + {{\rm{P}}_{\rm{M}}}{{\rm{P}}_{\rm{1}}}{{\rm{P}}_{\rm{2}}}{{\rm{P}}_3}{{\rm{P}}_4}{{\rm{P}}_5}{{\rm{P}}_6} \cr & + {{\rm{P}}_{\rm{M}}}{{\rm{P}}_{\rm{1}}}{{\rm{P}}_{\rm{2}}}{{\rm{P}}_3}{{\rm{P}}_4}{{\rm{P}}_5}{{\rm{P}}_6}{{\rm{P}}_7} + {{\rm{P}}_{\rm{M}}}{{\rm{P}}_{\rm{1}}}{{\rm{P}}_{\rm{2}}}{{\rm{P}}_3}{{\rm{P}}_4}{{\rm{P}}_5}{{\rm{P}}_6}{{\rm{P}}_7}{{\rm{P}}_8} \cr & + {{\rm{P}}_{\rm{M}}}{{\rm{P}}_{\rm{1}}}{{\rm{P}}_{\rm{2}}}{{\rm{P}}_3}{{\rm{P}}_4}{{\rm{P}}_5}{{\rm{P}}_6}{{\rm{P}}_7}{{\rm{P}}_8}{{\rm{P}}_9} \cr & + {{\rm{P}}_{\rm{M}}}{{\rm{P}}_{\rm{1}}}{{\rm{P}}_{\rm{2}}}{{\rm{P}}_3}{{\rm{P}}_4}{{\rm{P}}_5}{{\rm{P}}_6}{{\rm{P}}_7}{{\rm{P}}_8}{{\rm{P}}_9}{{\rm{P}}_{10 + }}/(1 - {{\rm{P}}_{{\rm{10 + }}}}) \cr}$$

### Study population

This article utilizes the 2015 Afghanistan Demographic and Health Survey [23] as the dataset. The total sample size, weighted for representativeness, was 21,324. The use of sample weights helps to ensure accurate estimates for the population of Afghanistan as a whole.

### Measurements

This paper uses Parity Progression Ratios (PPRs) to determine the Total Fertility Rate (TFR). PPRs are a measure of fertility that provide information on the likelihood of women giving birth to their next child, given that they have already had a certain number of children [28]. PPRs are calculated by dividing the number of live births during a specific time period by the number of live births in the preceding time period. Total Fertility Rate (TFR) is a measure of the average number of children that would be born to a woman over her lifetime if she were to experience the current age-specific fertility rates throughout her reproductive years [6]. Closed birth intervals (CBI) are the time duration between the birth of one child and the next. The predictor variable is self-reported experiences of abusive behavior or actions perpetrated by husbands/partners against women aged 15–49 living in the community. The 2015 Afghan Demographic and Health Survey included a series of questions related to physical, emotional, and sexual assault. The physical violence section involves asking about actions that hurt or harm women, such as pushing, shaking, throwing objects, hitting, twisting arms, pulling hair, punching, physically assaulting, trying to strangle or burn, and assaulting or intimidating with a weapon such as a knife. The sexual violence section of the survey includes questions about exerting physical force to make a woman have sex when she does not want to. The emotional violence section includes inquiries about saying or doing something to humiliate women in public, making threats to injure or damage them or their loved ones, and causing them to feel inferior about themselves. The answers were categorized as “yes” if the situation had ever been experienced and “no” if it had not. The independent variable in this study is Intimate Partner Violence, which includes the physical, sexual, and emotional dimensions of violence targeted toward women.

### Covariates

The selection procedure for controlling variables began with bivariate analysis, using the Chi-squared test (χ2) (see Supplementary Table 1). For the multivariate analysis, Variables that were incorporated in the analysis had a P-value below 0.25, which is a commonly used cutoff point in the literature and has been well-established in previous research [29]. Hence, in this study, certain factors such as place of residence, education level, wealth index, and the total number of years of education attained by the husband/partner were included as covariates to regulate the influence of husbands’ violence against women on fertility.

## Results

**Fig. 1:**
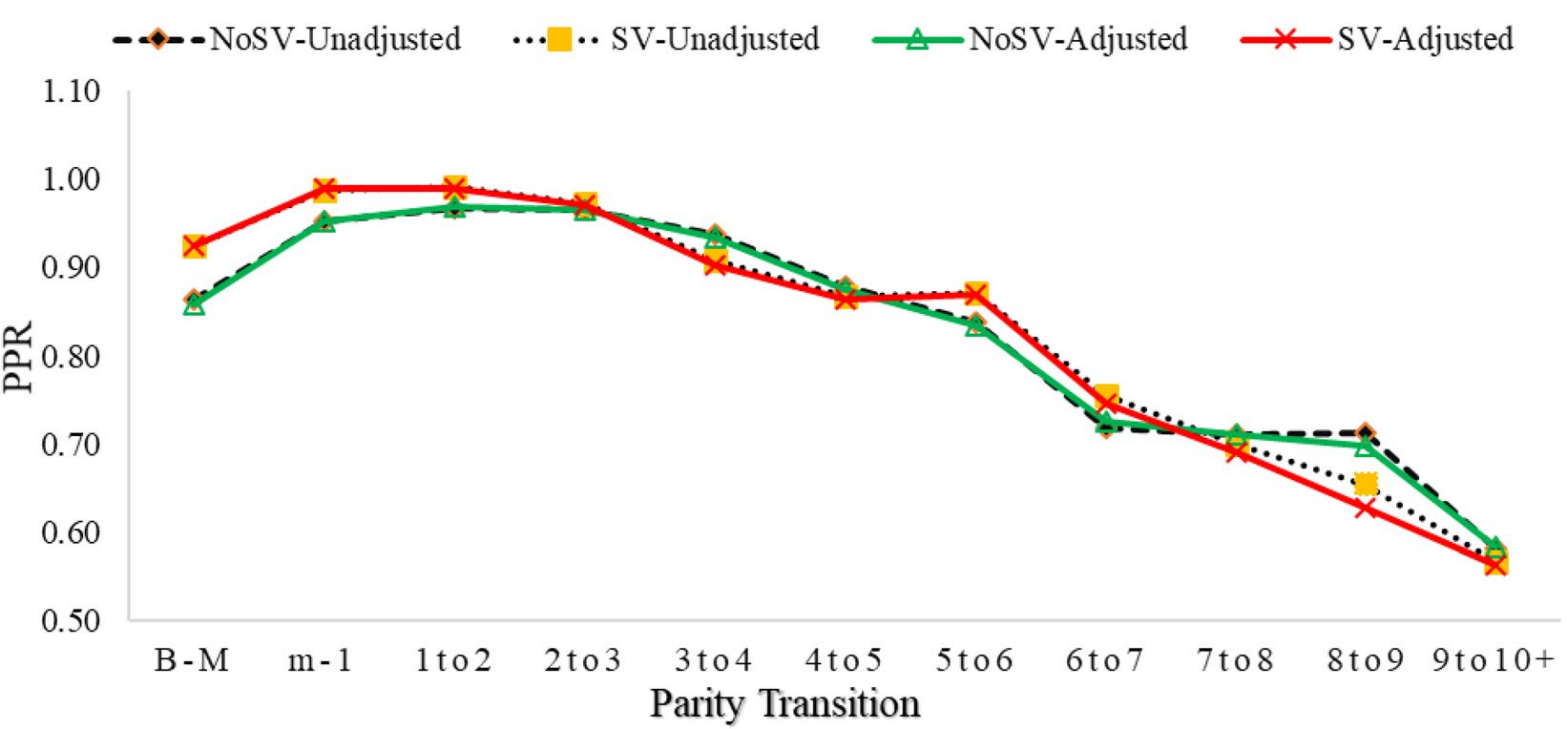
Comparison of parity progression ratio (PPR) by the experience of spousal violence (see Supplementary Table 2)

Figure 1 displays a comparison of the PPR between women who have experienced SV and those who have not. The figure presents two categories, “No SV” and “SV”, indicating the presence or absence of SV experience among women.

The graph displayed in Fig. 1 indicates that women who have experienced SV are more likely to progress to the next parity than women who have not faced spousal violence, particularly at the lower and middle parity stages. Additionally, the graph illustrates that women who have experienced SV tend to start childbearing at a faster pace (with higher initial PPRs) and continue to do so, while women who have not experienced SV tend to progress to higher parities at a slower pace in their initial reproductive years. Women who have not experienced SV appear to catch up to women who have experienced SV in middle parities (3–4, 4–5), but women who have experienced SV surpass them again at higher parities (5–6, 6–7). In the highest parities (7–8, 8–9, 10+), women who have not experienced SV are again ahead of women who have experienced SV.

The figure also shows that women with SV experience may have slightly higher PPR values, especially in certain intervals of parity progression. However, the differences between the two groups are not substantial, and PPR values are generally high for both groups.

**Fig. 2:**
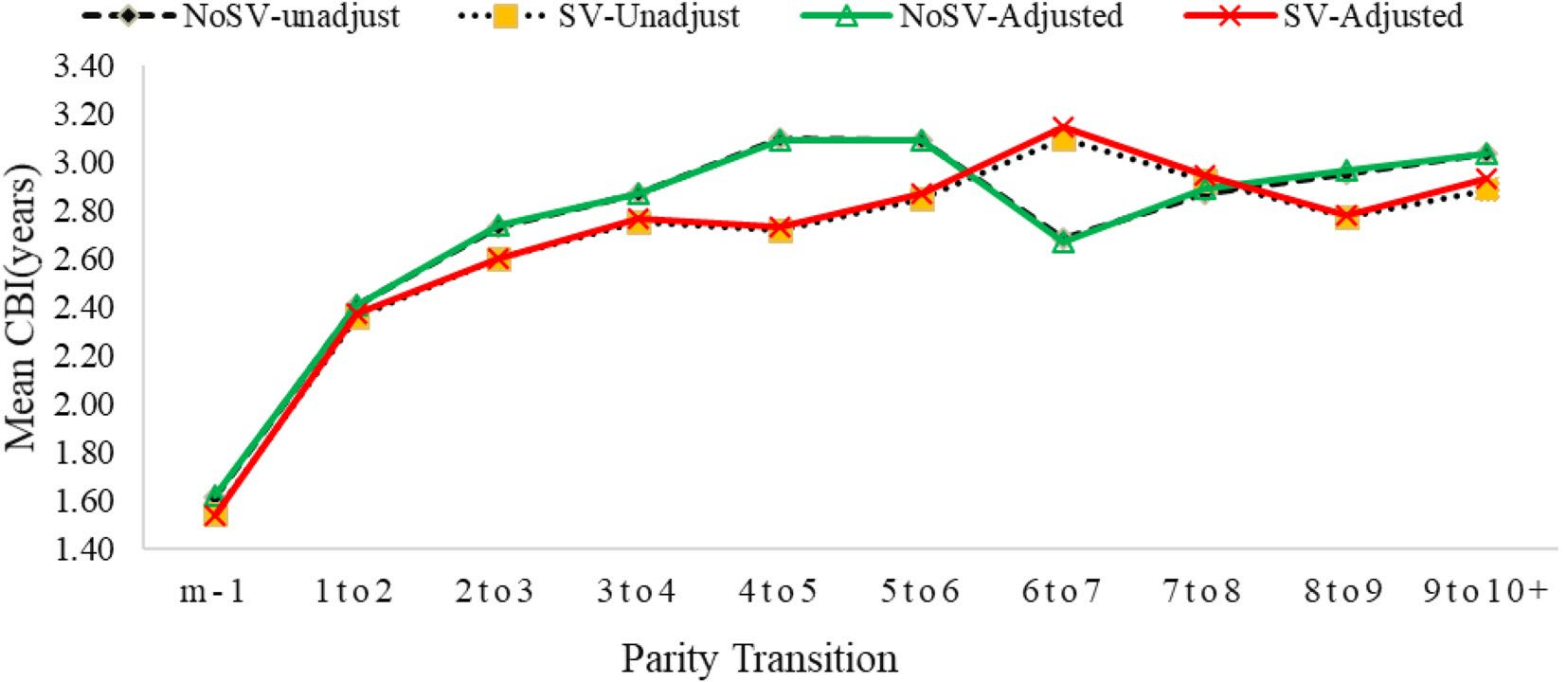
Comparison of mean closed birth intervals (CBI) among different birth orders by the experience of spousal violence (see Supplementary Table 3)

Figure 2 shows that the mean CBI values tend to increase as birth order increases, indicating that the length of time between births tends to increase as women have more children. The mean CBI values are generally similar for women with and without SV experience, with only small differences between the two groups. However, for some birth orders, such as the fourth and fifth child, the mean CBI values for women with SV experience are slightly lower than those for women without SV experience, especially in the unadjusted analysis.

The Figure also shows that the adjusted mean CBI values tend to be slightly higher than the unadjusted mean CBI values for both groups, suggesting that the confounding factors have some impact on the CBI values. However, the differences between the adjusted and unadjusted values are relatively small.

Table 1 displays both adjusted and unadjusted estimates of TFR based on the experience of partner violence. The data in the table shows the TFR estimates for each category, both adjusted and unadjusted. The unadjusted TFR for women who experienced SV is 7.0, which is higher than the unadjusted TFR for women who did not experience SV (6.2). However, after adjusting for other factors, the TFR for women who experienced SV decreases slightly to 6.9, while the TFR for women who did not experience SV remains the same at 6.2. This suggests that women who experienced SV may have a slightly higher TFR than those who did not, but the difference is not significant after adjusting for other factors.

**Table 1:** TFR based on spousal violence experience, a comparison of adjusted and unadjusted measures

		TFR
Experience violence		
SV	Unadjusted	7.0
	Adjusted	6.9
No SV	Unadjusted	6.2
	Adjusted	6.2

## Discussion

The findings of this study suggest that SVAW may have an impact on reproductive outcomes, including PPR, CBI, and TFR. Women who have experienced SV appear to reproduce more quickly and advance to higher parities faster than women without SV experience, especially at lower and middle parity steps. However, the differences between the two groups are generally small, and women without SV experience catch up at middle parities and surpass women with SV experience at the highest parities. The mean CBI values are generally similar for women with and without SV experience, with only small differences between the two groups. Previous studies have reported that IPV was found to be significantly associated with experiencing parenthood at an earlier age compared to those who have not experienced IPV [30]. This is likely because women who start childbearing at a younger age and a shorter distance from marriage will generally have more children than women who postpone childbearing [31]. Additionally, women’s experience of IPV is linked to a shorter duration between successive pregnancies [32].

The calculated TFR has been adjusted, revealing that women who have experienced SV have a slightly higher TFR compared to those who have not, even after accounting for potential confounding factors. This finding is consistent with previous research on the association between IPV and fertility. The study’s results establish SVAW as one of the unexplained factors contributing to persistently high fertility rates, particularly in countries like Uganda, as highlighted by Frade and Odimegwu (2018) [13]. These findings are also consistent with other studies. For instance, Stieglitz et al. (2018) observed that IPV predicts higher marital fertility among the Tsimané forager-horticulturalists of Bolivia [11], while Odimegwu et al. (2015) found that women who experienced domestic violence had elevated fertility rates in sub-Saharan Africa [12]. Furthermore, Wilson-Williams et al. (2008) explored the impact of domestic violence on contraceptive use in a rural Indian village, emphasizing the connection between violence and limited fertility decision-making [33].

The impact of SVAW on fertility outcomes can be understood through trauma theory. Trauma associated with SVAW can result in physical and psychological health problems, such as depression, anxiety, and post-traumatic stress disorder (PTSD), which can in turn affect fertility outcomes [34, 35]. Additionally, individuals who have experienced trauma may face barriers in seeking reproductive healthcare and accessing contraception, further exacerbating the impact of SVAW on fertility [36–39].

According to evolutionary theory, violence against women can be attributed to the need for men to control and limit women’s reproductive choices to ensure their investment in the production of future generations [40]. This is rooted in the fact that women are certain of their biological connection to their offspring, while men are not. As a result, men may employ various strategies to control women, as their reproductive contribution is perceived to be of greater value [40].

In certain contexts, men may have a larger ideal family size (IFS) compared to women due to lower investment costs per child [41, 42]. For example, in Afghanistan, unmarried women have an average IFS of 5.6, while men desire 6.2 children [22]. However, women with greater decision-making authority tend to align their fertility ideals with modern trends, which involve having fewer children [43].

Reproductive Coercion and Abuse (RCA) theory focuses on intentional efforts to manipulate an individual’s reproductive decisions, often perpetrated by intimate male partners [44]. This coercion can take various forms, including forced pregnancy, contraceptive obstruction, and pregnancy outcome management [45]. Existing literature has established the relationship between RCA, IPV, unwanted pregnancies, and increased fertility [11, 44, 46–53]. If husbands exert coercive control over their wives’ reproductive choices, as suggested by the SVAW explanation, it is possible that husbands’ higher ideal family size may result in higher fertility than desired by their wives. Alternatively, husbands may influence their wives to adjust their ideal number of children to meet their own preferences [11, 54]. Furthermore, family size can be influenced by the frequency of sexual intercourse, which women may have limited control over [41].

Women who experience violence may face challenges in accessing contraception due to their partner’s control over their reproductive choices [55]. This can include preventing them from using contraception, sabotaging their birth control methods, or coercing them into unprotected sex. Limited control over reproductive decisions puts women at a higher risk of unwanted fertility, unintended pregnancies, premature birth, miscarriage, and a perception of higher fertility than desired [52, 56–60].

In Afghanistan, having children is often viewed as a religious and cultural duty, and having a larger family is seen as a sign of wealth and prestige in many communities. Women who experience violence may feel pressured to have children to fulfill these cultural or social expectations, which can lead to a perception of higher fertility.

Afghanistan is currently experiencing a social crisis characterized by poverty, illiteracy, inadequate healthcare funding, lack of infrastructure, and ethnic and religious discrimination. These factors have contributed to a state of societal collapse. Afghan women are disproportionately affected by these challenges, as cultural and political barriers, as well as insecurity, hinder their access to education and empowerment. They often face illiteracy, discrimination, confinement to their homes, punishment, neglect, abandonment, torture, and even maternal mortality. To address the higher TFR in Afghanistan, it is crucial to implement effective policies and programs that promote women’s education and empowerment, improve access to family planning services, enhance maternal and child healthcare, and challenge the social and cultural norms that perpetuate spousal violence against women. Additionally, community-based interventions involving men and boys in promoting gender equality and preventing violence against women can play a significant role in reducing SVAW and improving the health outcomes of mothers and children.

## Conclusion

It is important to consider the potential impact of SVAW on women’s reproductive decision-making, access to reproductive healthcare, and autonomy. Addressing SVAW and promoting reproductive health and rights are crucial for ensuring that women can make informed decisions about their reproductive lives and have control over their bodies and fertility. Health professionals and policymakers should prioritize the provision of comprehensive services and support for women who have experienced SV and work towards creating a safe and supportive environment for all women to access reproductive healthcare and exercise their reproductive rights.

SVAW not only causes immediate harm to the victims but also has far-reaching consequences for the whole family system and society. These consequences are interconnected like chain links and can cause serious damage. In light of this, the study highlights the need for policies and programs aimed at regulating fertility and reproductive rights in Afghanistan to pay attention to the social values and norms that foster gender inequality and violence against women. By addressing these underlying factors, policies and programs can help prevent the harm caused by SVAW and promote reproductive health and rights for women in Afghanistan.

Furthermore, the findings underscore the importance of promoting reproductive health and rights. Women who have undergone SVAW may encounter obstacles in obtaining reproductive healthcare services, and it is vital to guarantee that they receive the necessary resources and assistance to make informed choices about their reproductive health. The results of the study also emphasize the significance of further investigating the intricate connection between SVAW and reproductive outcomes and identifying effective ways to enhance reproductive health for women exposed to SVAW.

### Limitation of the study

The main limitation of this research is the calculation of standard errors of estimates, which typically involves the use of the jackknife method. However, this method requires a significant amount of super-fast computer time, often taking several months to complete.

To address the limitations of inferring changes in time trends based on cross-sectional studies, it is recommended that future research incorporate longitudinal data. Additionally, replicating this study in diverse cultural and social contexts can help determine if similar patterns exist beyond the original study setting. This approach would establish the generalizability of the findings and provide a more comprehensive understanding of the relationship between spousal violence and reproductive outcomes.

### Electronic supplementary material

Below is the link to the electronic supplementary material.

Supplementary Material 1

## Data Availability

Data for this study were sourced from Demographic and Health Surveys (DHS) and are available here: http://dhsprogram.com/data/available-datasets.cfm.

## Abbreviations

DHS: Demographic and Health Survey
SVAW: Spousal Violence Against Women
IPV: Intimate Partner Violence
PPRs: Parity Progression Ratios
TFR: Total Fertility Rate
CBI: Closed birth intervals
