# Supplementary material for: Investigating the relationship between spousal violence against women and total fertility rate in Afghanistan
Source: BMC Public Health. 2024 May 31;24:1463. doi: 10.1186/s12889-024-18944-6 (PMC11143615; doi:10.1186/s12889-024-18944-6)
Supplement: Supplementary file 1 — Supplementary Material 1 [file 12889_2024_18944_MOESM1_ESM.docx]

**SUPPLEMENTARY INFORMATION**

**Investigating the Relationship between Intimate Partner Violence and Total Fertility Rate in Afghanistan**

Mehri Shams Ghahfarokhi, Ph.D.

Assistant Professor, University of Isfahan, Department of Social Sciences, Isfahan, Iran.

E-mail: m.shams@ltr.ui.ac.ir

ORCID ID: [https://orcid.org/0000-0003-3743-8543](file:///F:\maghale-salmand-tannha\(https:\orcid.org\0000-0003-3743-8543)

Supplementary Table 1. Distribution of the independent variable and control variables according to the dependent variable (having children) along with the chi-square test results in the five years before Afghanistan Demographic and Health Survey, 2015^a^

| Variable | Having children in the last five years | | P value^b^ |
| --- | --- | --- | --- |
|  | No (n = 101678) | Yes (n = 29613) | <0.001 |
| **women have experienced any form of intimate partner violence (physical, emotional, and sexual assault)** |  |  |  |
| No (n = 46617) | 36402 (78.09) | 10215 (21.91) | <0.001 |
| Yes (n = 48586) | 36830 (75.80) | 11756 (24.20) |  |
| **physical intimate partner violence** |  |  |  |
| No (n = 50154) | 39071(77.90) | 11083 (22.10) | <0.001 |
| Yes (n = 45074) | 34184 (75.84) | 10890 (24.16) |  |
| **emotional intimate partner violence** |  |  |  |
| No (n = 64515) | 49831(77.24) | 14684 (22.76) | <0.001 |
| Yes (n = 30703) | 23415 (76.26) | 7288 (23.74) |  |
| **sexual intimate partner violence** |  |  |  |
| No (n = 86828) | 66879 (77.02) | 19949 (22.98) | <0.022 |
| Yes (n = 8245) | 6259 (75.91) | 1986 (24.09) |  |
| **Age** |  |  |  |
| 15-19 (n = 18366) | 15144 (82.46) | 3222 (17.54) | <0.001 |
| 20-24 (n = 29742) | 19990 (67.21) | 9752 (32.79) |  |
| 25-29 (n = 26038) | 18149 (69.70) | 7889 (30.30) |  |
| 30-34 (n = 18755) | 14136 (75.37) | 4619 (24.63) |  |
| 35-39 (n = 17782) | 14904 (83.82) | 2878 (16.18) |  |
| 40-44 (n = 13165) | 12120 (92.06) | 1045 (7.94) |  |
| 45-49 (n = 7443) | 7235 (97.21) | 208 (2.79) |  |
| **Residence** |  |  |  |
| Urban (n = 30689) | 23812 (77.59) | 6877 (22.41) | 0.483 |
| Rural (n = 100602) | 77866 (77.40) | 22736 (22.60) |  |
| **Women's education** |  |  |  |
| No education (n = 112663) | 87147 (77.35) | 25516 (22.65) | <0.001 |
| Primary (n = 8599 ) | 6624 (77.03) | 1975 (22.97) |  |
| Secondary (n = 7748) | 6031 (77.84) | 1717 (22.16) |  |
| Higher (n= 2281) | 1876 (82.24) | 405 (17.76) |  |
| **Education of husbands** |  |  |  |
| No education (n = 75557) | 58601 (77.56) | 16956 (22.44) | <0.001 |
| Primary (n = 17384) | 13313 (76.58) | 4071 (23.42) |  |
| Secondary (n = 28821) | 22334 (77.49) | 6487 (22.51) |  |
| Higher (n = 9164) | 7163 (78.16) | 2001 (21.84) |  |
| **Wealth index** |  |  |  |
| Poorest (n = 25125) | 19728 (78.52) | 5397 (21.48) | <0.001 |
| Poorer (n = 30211) | 23320 (77.19) | 6891 (22.81) |  |
| Middle (n = 28664) | 21924 (76.49) | 6740 (23.51) |  |
| Richer (n = 27801) | 21360 (76.83) | 6441 (23.17) |  |
| Richest (n = 19490) | 15346 (78.74) | 4144 (21.26) |  |

^a^ The frequencies are based on the expanded data

^b^ P values were calculated using a Chi-squared test (χ^2^)

Supplementary Table. 2. Comparison of parity progression ratio (PPR) by the experience of spousal violence

|  | **NoSV-Unadjusted** | **SV-Unadjusted** | **NoSV-Adjusted** | **SV-Adjusted** |
| --- | --- | --- | --- | --- |
| B-M | 0.86 | 0.93 | 0.86 | 0.92 |
| m-1 | 0.95 | 0.99 | 0.95 | 0.99 |
| 1to2 | 0.97 | 0.99 | 0.97 | 0.99 |
| 2to3 | 0.97 | 0.97 | 0.97 | 0.97 |
| 3to4 | 0.94 | 0.91 | 0.93 | 0.90 |
| 4to5 | 0.88 | 0.87 | 0.88 | 0.86 |
| 5to6 | 0.84 | 0.87 | 0.84 | 0.87 |
| 6to7 | 0.72 | 0.75 | 0.73 | 0.75 |
| 7to8 | 0.71 | 0.70 | 0.71 | 0.69 |
| 8to9 | 0.71 | 0.65 | 0.70 | 0.63 |
| 9to10+ | 0.58 | 0.57 | 0.58 | 0.56 |

Supplementary Figure. 1 Illustrates the comparison of the parity progression ratio (PPR) based on the experience of spousal violence, as shown in Table 2.

Supplementary Table 3. Comparison of mean closed birth intervals (CBI) among different birth orders by the experience of spousal violence

|  | NoSV-Unadjusted | SV-Unadjusted | NoSV-Adjusted | SV-Adjusted |
| --- | --- | --- | --- | --- |
| m-1 | 1.62 | 1.55 | 1.62 | 1.54 |
| 1to2 | 2.41 | 2.36 | 2.41 | 2.37 |
| 2to3 | 2.73 | 2.60 | 2.74 | 2.60 |
| 3to4 | 2.87 | 2.75 | 2.87 | 2.77 |
| 4to5 | 3.10 | 2.72 | 3.09 | 2.73 |
| 5to6 | 3.09 | 2.85 | 3.09 | 2.87 |
| 6to7 | 2.69 | 3.10 | 2.67 | 3.14 |
| 7to8 | 2.87 | 2.93 | 2.89 | 2.94 |
| 8to9 | 2.96 | 2.77 | 2.97 | 2.78 |
| 9to10+ | 3.03 | 2.89 | 3.04 | 2.93 |

Supplementary Figure. 2. Illustrates the comparison of mean closed birth intervals (CBI) among different birth orders by the experience of spousal violence, as shown in Table 3
